# Deep learning-based early warning of tailings storage facility instability using Sentinel-1 and Radarsat-2 InSAR calibrated with a geomechanical model

**DOI:** 10.1038/s41598-026-51005-z

**Published:** 2026-07-20

**Authors:** Maral Bayaraa, Brian Sheil, Cristian Rossi

**Affiliations:** 1 Engineering Science department, University of Oxford, Oxford, UK; 2 Laing O’Rourke Centre for Construction Engineering and Technology, University of Cambridge, Cambridge, UK; 3 Earth Observation Programmes (EOP-SME), European Space Agency, Noordwijk, The Netherlands

**Keywords:** Engineering, Natural hazards, Solid Earth sciences

## Abstract

This study advances the science and application of Interferometric Synthetic Aperture Radar (InSAR) for monitoring tailings storage facilities (TSFs) by integrating multi-scale and multi-source satellite data with geomechanical modelling and deep learning to detect ground movement precursors to failure. A novel calibration strategy combining geomechanical finite element (FE) predictions with InSAR measurements is introduced to dissect the origins and expressions of known InSAR limitations related to phase unwrapping. By investigating different InSAR processing algorithms along with data from both commercial (Radarsat-2) and public (Sentinel-1) sensors, the study demonstrates the extent to which these limitations can be overcome. During periods of rapid or non-linear deformation, such as buttress construction over the TSF, Persistent Scatterer InSAR (PS-InSAR) time series show more sensitivity to phase unwrapping errors, making phase ambiguities more readily observable. The extent to which such ambiguities can be identified and calibrated using physics-based finite element model predictions is demonstrated in this study. In contrast, Intermittent Small Baseline Subset (ISBAS) processing reduces the visible expression of phase ambiguities through inherent signal averaging (i.e. multi-looking), while maintaining a higher spatial density of deformation measurements. An uncertainty analysis for both InSAR approaches links accuracy to SAR signal distortions and uncertainties in FE model parameters. Finally, the capabilities of the deep learning early-warning framework capable of ingesting multi-source InSAR datasets, including different sensors, geometries and decomposed motion components are presented. Embedding-based integration of line-of-sight and decomposed Radarsat-2 (RS2) InSAR data reveals that orbit configuration and resolution directly influence predictive performance. Specifically, the horizontal deformation components provide the clearest precursors to failure, outperforming line-of-sight and vertical motion components. The combined use of RS2-InSAR, Sentinel-1 PS-InSAR, Sentinel-1 ISBAS, FE modelling and deep learning offers a promising framework for TSF monitoring. This integrated approach promises to reduce uncertainties inherent in individual methods and to significantly improve the ability to reduce risks of environmental and economic losses.

## Introduction

Investors, governments and civil societies are advocating for greater transparency on the global social and environmental risks associated with mine tailings storage facilities (TSFs)^1^. The recent rise in serious failures^2^, have resulted in severe socio-environmental tragedies, such as the Brumadinho failure of 2019, Brazil^3^ and more recently, the Jagersfontein failure in 2022, South Africa^4^. In response, a group of ethical investors^5^ launched the largest disclosure initiative to date. This represents a pioneering effort setting a precedent for investor-driven demands for corporate accountability on mining risk. However, the initiative ultimately accounted for only about 30$\%$ of global commodity production, with many companies seemingly reluctant to disclose their TSF risks^6^. Even among operators willing to disclose, many were unable to provide complete engineering records. Among the 1743 TSFs surveyed, 257 facilities (15 $\%$) lacked full documentation^6^. These gaps reflect the inherent complexities faced by mine operators, as TSFs are large, long term projects spanning decades, often passing multiple mine ownerships and political parties.

A key challenge in improving TSF risk lies in the complexity of data availability. On one end of the spectrum, government, civil society and investor stakeholders lack the same level of access to TSF data as mining companies do. Yet even for mine operators, developing rigorous geotechnical models requires extensive monitoring campaigns, which are both resource-intensive and subject to uncertainty. The limitations of even the most comprehensive geotechnical approaches were illustrated by the failure of the Cadia TSF, which occurred on the 9th of March 2018, in South-West Australia. The post-failure investigations by Jefferies et al. (2019) provide one of the most extensive, open geotechnical insights on TSF failures to date^7^. These investigations revealed that, despite rigorous monitoring, the TSF failed because it was constructed over a previously unidentified weathered foundation bedrock (’the FRV Unit-A’), which had not been incorporated into pre-failure models^7^. The absence of this knowledge introduced considerable uncertainty into geotechnical and safety analyses, demonstrating the extent to which even state-of-the-art techniques are vulnerable to such unknowns.

In this context, remote sensing technologies, particularly Interferometric Synthetic Aperture Radar (InSAR)^8,9^, are increasingly recognised as valuable complements to conventional geotechnical monitoring. InSAR analysis enables the detection of millimetric-scale ground movements from satellite constellations hundreds of kilometres in space. For geotechnical engineers, InSAR has the potential to provide an independent dataset that complements more localised ground-based monitoring and modelling, and has shown promising results for a variety of critical infrastructure, such as water dams^10–13^, underground construction activities^14^, ground de-watering^15^, railway-^16^ and road-embankments^17^ and bridges^18,19^.

Recent studies leveraged InSAR for back-analysis of failed TSFs, such as the Brumadinho^20,21^ and Cadia failures^22–25^. A key research question has been whether InSAR can detect precursory indications of structural failure preceding these failures. The opportunistic nature of InSAR has been demonstrated to provide measurements in areas not previously considered critical, which can result in insights that may otherwise be overlooked^21–24^. Its rich spatial and temporal measurement has proven particularly valuable for capturing evolving deformation dynamics. During the period immediately preceding the Cadia TSF failure, ground-based instrumentation had been removed, leaving InSAR as the only available source of insight. While demonstrating the complementary role of InSAR alongside geotechnical modelling and ground-based instrumentation, Bayaraa et al. (2024)^25^ also highlighted the impact of InSAR’s known limitations for TSF monitoring. For example, geotechnical model predictions diverged from InSAR measurements following buttress construction, likely due to the reduced capability of InSAR in capturing rapid deformation signals.

Data-driven approaches have recently shown considerable promise for extracting decision-ready insights from the large volumes of measurements generated by InSAR, with applications spanning deformation detection, classification and prediction across a range of geophysical and infrastructure contexts. In particular, deep learning methods have been applied to identify deformation signals associated with volcanic activity^26^, underground infrastructure and water or gas extraction^27–29^, airports^30–32^, roads^33^, and geological faults^34,35^. Both sequential-data-based recurrent neural networks and image-based convolutional neural networks have been explored in this context. In some studies, InSAR deformation time series are treated as purely sequential data^32^, where individual deformation measurements are modelled independently based on their temporal evolution. While effective for certain applications, such approaches typically provide limited representation of spatial relationships between neighbouring measurements. Image-based approaches, in contrast, explicitly exploit spatial context but often require additional preprocessing steps, such as spatial gridding or synthetic data generation, to accommodate the irregular and sparse spatial distribution of InSAR observations^27,31^. These characteristics motivate alternative modelling strategies, such as embedding-based deep learning approaches^36^, that can directly accommodate the native spatio-temporal structure of InSAR.

The primary objective of this study is to demonstrate how physics-based finite element (FE) modelling can be explicitly integrated with multi-source InSAR measurements and deep learning to guide the interpretation, correction and use of InSAR time series for tailings storage facility monitoring. Rather than proposing a single automated unwrapping algorithm or prescriptive calibration recipe, the study establishes how these complementary approaches can be combined to address known limitations of InSAR and improve early-warning capability. This study advances the science and application of InSAR for TSF monitoring by (1) systematically dissecting the origins and expressions of known InSAR limitations, (2) demonstrating that these limitations are not inherent to InSAR, but can be overcome through algorithmic refinements and by integrating multi-scale, multi-source data, highlighting that certain InSAR approaches may be better suited to TSFs; (3) evaluating the influence of satellite sensor characteristics, such as resolution and orbit configuration, on monitoring performance, and (4) presenting, an integration of multi-source InSAR data into an early warning framework based on Embedded Entities in Deep Learning (EE-DL), revealing how orbit and resolution directly influence predictive performance. For stakeholders lacking access to site-specific geotechnical data, these advances promise an unprecedented, remote insight into TSF behaviour and risk.

## Results

The results demonstrate how multi-source and multi-scale data integration advances InSAR-based monitoring of TSFs. Experiments using different InSAR algorithms and data from both open-access Sentinel-1 (S1) and commercial Radarsat-2 (RS2) sensors reconcile the extent to which known InSAR limitations, particularly phase ambiguities, can be overcome, showing that these challenges are not inherent to the method itself. Phase-ambiguity correction is validated through independent data sources from ground-based measurements and geomechanical model predictions.

The integration of InSAR, FE modelling and deep learning demonstrates a clear synergy in which each component compensates for limitations of the others. Physics-based FE modelling provides mechanistic constraints on deformation magnitude, directionality and temporal behaviour. It informs the interpretation and correction of InSAR time series, particularly in resolving phase ambiguities and aligning deformation components into physically meaningful directions. InSAR, in turn, provides spatially dense deformation measurements that is often lacking in ground-based monitoring or modelling alone, enabling independent validation and stress-testing of FE predictions. These corrected and physically consistent InSAR deformation signals are then subsequently ingested by the deep learning framework, enhancing its ability to distinguish anomalous deformation associated with instability from background consolidation behaviour. Together, this integrated multi-disciplinary approach improves the robustness and interpretability of anomaly detection compared to any single method applied in isolation. Moreover, the value of multi-source and multi-scale InSAR data and its seamless integration into the early warning deep learning algorithm further demonstrates the impact of using both line-of-sight and decomposed motion components, advancing AI-driven predictive monitoring for geotechnical systems.

The key differences between S1 and RS2 reflect their sensor characteristics, particularly spatio-temporal resolutions and data accessibility. S1 provides medium-resolution, globally available open-source data, whereas RS2 offers high-resolution commercial data with non-uniform global coverage. In general, the characteristics of S1 data are more consistent than those typically associated with commercial SAR data. For example, although the S1 data is approximately five times coarser than the RS2 data available over Cadia, RS2 can also operate in different acquisition modes. This means, RS2 may not always provide the fine spatial resolution that is available over Cadia. Moreover, S1 over Cadia is limited to the descending orbit, whereas RS2 are available from both ascending and descending orbits. This restricts the ability to decompose S1 into its vertical and horizontal deformation components, in contrast to RS2, as outlined in Tables 1 and 2. Transitioning from S1 to RS2 represents an order-of-magnitude rise in data volume, enabling tests of methodological adaptations required for scaling predictive approaches. This scalable integration is critical to continental initiatives such as the European Ground Motion Service^37,38^, which illustrate the feasibility of large-area InSAR monitoring and signal that global, near-real-time ground-deformation surveillance is within reach.

Although the Cadia TSF is a specific case study, its failed sections represent the most failure-prone construction type, the upstream method. Upstream TSFs are roughly twice as likely to experience stability issues as downstream facilities and six times more likely than dewatered “dry-stack” designs^6^. Despite recent bans in some countries such as Chile and Brazil^39^, many upstream TSFs remain operational worldwide, highlighting the urgency of scalable, remote early-warning systems.

### Correcting and explaining the source of phase ambiguities

From the basic characteristics of Synthetic Aperture Radar (SAR) waves, the maximum movement that can be detected per repeat cycle equals $\frac{\lambda }{4}$, where $\lambda$ is the radar wavelength^40^. Based on Itoh’s condition^41^, a wrapping threshold can be defined^40^. In the case of the C-band SAR sensors used in this study (S1 and RS2), the wrapping threshold equals $\frac{\lambda }{4} = 14$ mm. This means, if a measurement is close to the $\pm \frac{\lambda }{4}$ threshold between any two dates of acquisitions, then there is a risk of encountering phase ambiguity. For example, Reinders et al. (2021)^40^ tackled the likelihood of encountering phase ambiguities by considering alternative deformation solutions approaching the $\pm \pi$ threshold. Then, independent measurements from ground-based instrumentation (levelling) was used to select the true InSAR solution. In real world scenarios, the Itoh’s condition for phase unwrapping may not be fulfilled due to factors such as noise, fast deformation, or actual discontinuities between adjacent samples^41^. This can result in the incorrect multiples of $2 \pi$ being added or subtracted in the phase unwrapping process.

**Fig. 1:**
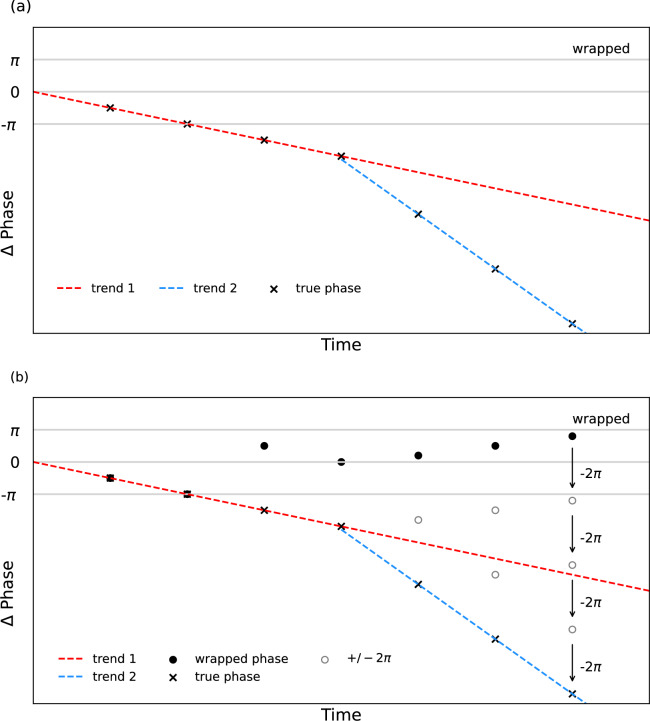

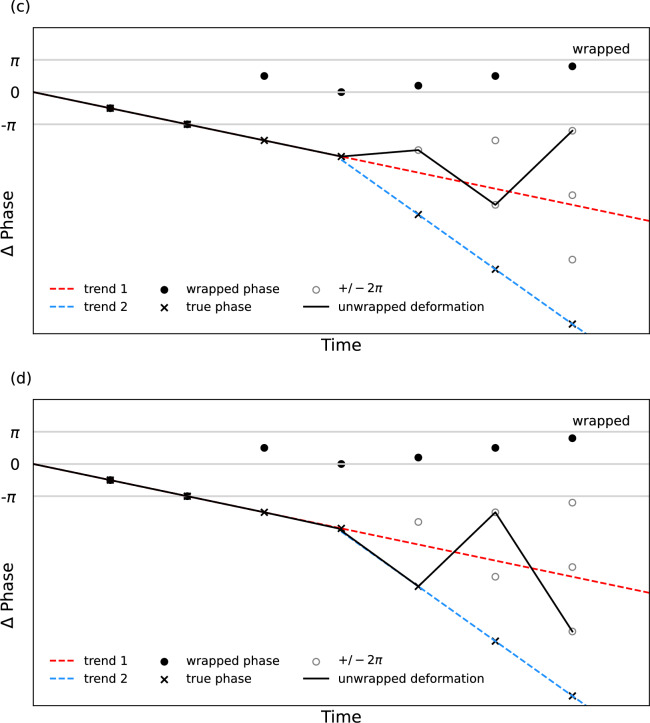
A demonstration of phase unwrapping using a linear deformation model. The true deformation trends are plotted in (**a**), where trend 1 changes to a steeper trend 2. The wrapped phases, confined within the $\pm \pi$ range represent what would be measured by InSAR, as shown in (**b**). To recover the true deformation trends from the wrapped phases, an unknown multiple of $2\pi$ needs to be subtracted. Potential unwrapping solutions are illustrated in (**c**) and (**d**), both of which represent examples of incorrect phase unwrapping.

Within the context of the Cadia TSF, the geotechnical model predictions from Bayaraa et al. (2024)^25^ suggest that the TSF deformation accelerates following the construction of buttresses. This change in the TSF deformation behaviour and its potential impact on phase unwrapping is explored in Fig. 1a–d. The true deformation of the TSF is simulated in Fig. 1a, where the trend changes over time from trend 1 to trend 2. For each measurement, multiples of $2\pi$ are added until the phases fall within the $\pm \pi$ range, as illustrated in Fig. 1b. The measurements within the $\pm \pi$ range correspond to the wrapped phases, which are the actual quantities observed with InSAR.

For instance in the PS-InSAR approach, an assumption of a linear deformation model is made, as represented by trend 1. The number of integer multiples of $2\pi$ that must be added to the true phases along trend 2 to obtain the wrapped phases is unknown. Given this uncertainty surrounding the exact solution, both plots in Fig. 1c,d provide potentially viable but incorrect solutions to phase unwrapping. In this example, it is not possible to resolve the phase ambiguity and find the true solution from InSAR alone without external data.

**Fig. 2:**
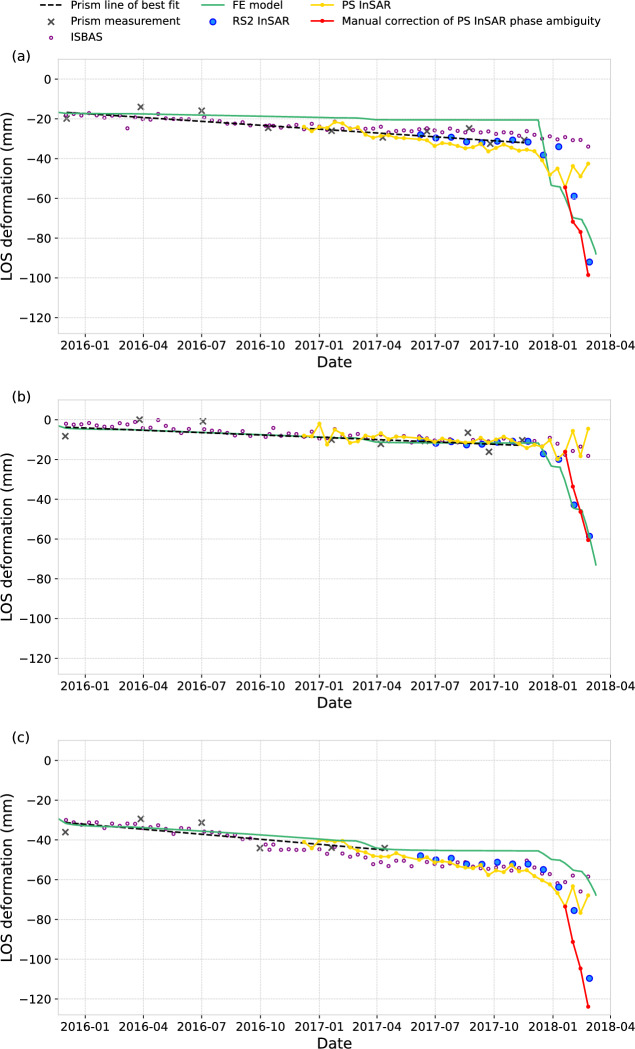
Temporal comparison of deformation in the satellite line-of-sight (LOS) from FE model predictions, ground-based prism measurements with descending S1-based ISBAS and PS- InSAR and RS2 InSAR for construction stages (**a**) 4, (**b**) 5 and (**c**) 7. Potential solutions to PS- InSAR phase ambiguity are proposed based on manual correction.

In the case of Cadia TSF, the geotechnical modelling results, prism measurements and RS2-InSAR data are available as external data for verifying S1 based ISBAS and PS-InSAR. The comparison is based on the locations of the ground-based prism instruments available within the failure area, as shown in the map in Fig. 11b. Figure 2a–c show the temporal comparison between FE predictions, prism measurements, RS2-InSAR, S1-based PS-InSAR and S1-based ISBAS at these locations. PS-InSAR displays high-magnitude irregularities following buttress construction, which are indicative of phase ambiguities arising from violations of the assumed linear deformation model. In contrast, the same assessment cannot be applied to ISBAS, as it does not exhibit clear phase unwrapping errors in the resulting time series. This difference reflects the fundamental characteristics of SBAS-type methods, which employ spatial and temporal averaging (or multi-looking) over distributed scatterers. Such averaging suppresses abrupt phase inconsistencies that would otherwise manifest as large jumps in the time series, but may also dampen true high-magnitude deformation signals.

PS-InSAR estimates deformation at individual scatterers and is therefore more sensitive to rapid or non-linear deformation that can violate unwrapping conditions and appear as the irregular phase jumps that can be seen in Fig. 2a–c. ISBAS, by contrast, incorporates averaging across pixels and interferograms, which reduces the visible expression of phase ambiguities. As a result, phase unwrapping issues in ISBAS are less readily detectable, making it more challenging to identify and correct such errors using external reference data.

Unfortunately, since the prisms were removed during the construction of the buttresses, it is not possible to use their measurements as ground-truth data for the period in which the phase ambiguities occur. Therefore, the FE model results and RS2-InSAR datasets may be used as reference for the number of multiples of $2\pi$ required for PS-InSAR phase ambiguity illustrated in Fig. 2a–c. The phase ambiguity correction presented here is case-specific and illustrative, demonstrating the feasibility of FE predictions and independent RS2-InSAR guided alignment rather than providing an independent validation of PS-InSAR performance. The development of a fully automated and generalisable phase unwrapping correction workflow is beyond the scope of the present study.

In these plots, the InSAR data agree relatively well with geotechnical model predictions up to the construction of buttresses. The buttresses were constructed in the slump area on 2017-12-09 and 2018-01-12 (YYYY-MM-DD), respectively. The phase ambiguity of PS-InSAR is addressed through subtraction of the following multiples of $2\pi$ from the last three measurements, 2018-02-01, 2018-02-13 and 2018-02-25: 1x$2\pi$, 1x$2\pi$, 2x$2\pi$, respectively. This FE guided phase-ambiguity calibration is implemented manually to demonstrate the feasibility of identifying and resolving phase ambiguities in PS-InSAR data. Phase ambiguity corrections are restricted to discrete integer multiples of 2$\pi$, consistent with the physical nature of phase wrapping and are applied only at time steps identified as susceptible to ambiguity based on deformation rates approaching the InSAR wrapping threshold.

The phase ambiguity corrected S1 PS-InSAR agrees well with both geotechnical FE predictions and high resolution RS2-InSAR measurements for dam stages 4 and 5 in Fig. 2a,b. However, in the case of dam stage 7 in Fig. 2c, the phase ambiguity corrected PS-InSAR agrees well with RS2-InSAR but not the geotechnical model results. The post buttress construction behaviour of PS- and RS2-InSAR data are similar to the behaviours observed for stages 4 and 5 in Fig. 2a,b. Interestingly, for TSF stage 7, both S1 PS-InSAR ambiguity-corrected data and RS2 InSAR exhibit a strong agreement with each other, but not with the FE model. The fact that deformation measurements from two independent InSAR processing methods using different types of SAR data agree is significant. However, given the uncertainties inherent in both InSAR and geotechnical modelling, it is challenging to resolve this discrepancy without external ground-truth data. If the FE model represents the truth, then the discrepancies may be attributed to factors such as distortions resulting from the interaction of the SAR signal relative to TSF geometry. For example, the steepness of the later dam stages such as stage 7 may make it more susceptible to the impacts of known radar distortions such as shadow or layover. Other factors such as the size of the spatial footprint of the dam stages being relatively small compared to that of the buttresses may be causing the SAR signal to reflect in ways that result in mixed signals from stage 7 and the buttresses.

**Fig. 3:**
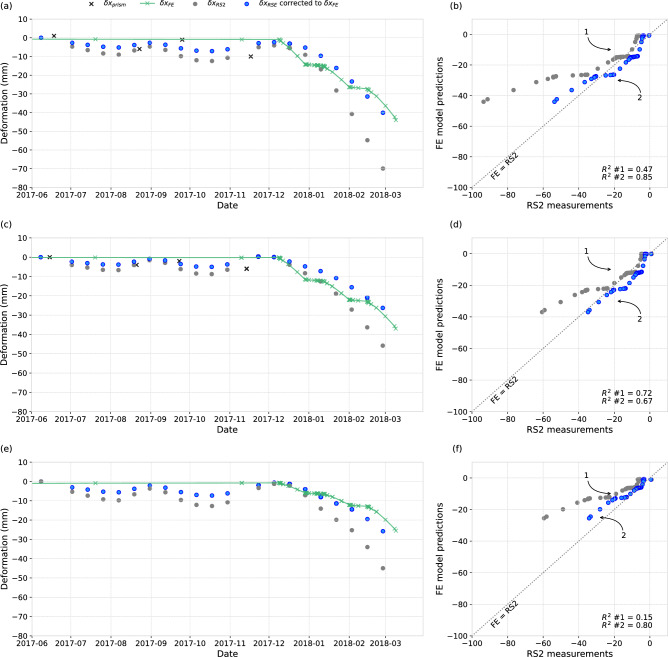
The horizontal deformation components ($\delta x$) for prisms, FE, RS2 are plotted. These plots represent the deformation measured at the following stages : (**a**) and (**b**) correspond to stage 4; (**c**) and (**d**) correspond to stage 5; and (**e**) and (**f**) correspond to stage 7. Since the horizontal measurements for RS2 and FE are in different directions, the RS2 has been adjusted to the same geometry as that of FE. The improvement resulting from the geometric correction is quantified in (**b**), (**d**) and (**f**) (right column); and the dotted line represents the line of perfect fit, where RS2 measurements equal FE model predictions.

On the other hand, if the S1 PS-InSAR and RS2-InSAR data represent the truth, then it may indicate that the FE model is not able to accurately capture the deformation of later dam stages such as stage 7. This can result from uncertainties in the modelling and parameter selection. As demonstrated in Bayaraa et al. (2024)^25^, the deformation signals of later dam stages are dominated by the deformation of the tailings, as they rest on thicker tailings. Therefore, this discrepancy may arise from uncertainties in the parameters controlling the mechanical response of the tailings in the FE model. For instance, the tailings are represented as single units for each TSF stage, which does not capture the fact that tailings properties, such as particle coarseness, vary horizontally^42–45^.

Both differences in SAR data spatial resolution, along with phase unwrapping errors, have been proposed as possible explanations for the large discrepancies in the measurement distributions observed between S1 PS-InSAR and RS2-InSAR. The successful alignment of S1 PS-InSAR deformation magnitudes with RS2-InSAR after phase ambiguity correction implies that pixel size differences have minimal impact. This indicates that the inability of S1 InSAR to capture large magnitude deformations preceding TSF failure is not an inherent limitation of the sensor, but rather a challenge that can be addressed through adjustments in InSAR processing and phase ambiguity correction.

The main sources of noise in SAR interferometry arise from sensor noise or from inadequate atmospheric phase screening. Phase unwrapping errors in InSAR data may therefore occur due to: (1) deformation approaching or exceeding the wrapping threshold, leading to an incorrect ambiguity number; (2) assumptions made in the unwrapping methodology, for example, in PS-InSAR, a linear deformation model may fail to capture non-linear deformation behaviour; and (3) signal noise. The simplified scenario illustrated in Fig. 1 does not account for complexities such as noise, which can disrupt the phase unwrapping process by being misinterpreted as large deformation signals.

### Impact of phase ambiguities on decomposed InSAR motions

The impact of phase ambiguities are further examined through their influence on the decomposed vertical and horizontal components of InSAR. Because S1 data over the Cadia TSF are available only from a descending orbit, motion decomposition is not feasible. Therefore, the decomposed RS2-InSAR motions are investigated along with ground-based prism measurements and geomechanical model predictions.

The horizontal components of RS2-InSAR, $\delta x_{RS2}$, projected to the horizontal motion direction of the FE predictions, $\delta x_{FE}$, are plotted in Fig. 3 for the following stages: (a) and (b) correspond to stage 4; (c) and (d) stage 5; and, (e) and (f) stage 7. The horizontal deformation through time is plotted in Fig. 3 (a), (c) and (e) (left column) and the improvements from the geometrical correction are quantified in Fig. 3 (b), (d) and (f) (right column). The coefficient of determination, $R^2$, is calculated between FE predictions and the line of best fit for the RS2-InSAR measurements, given their differing temporal sampling. Overall, the geometrical correction significantly improves the alignment, with $R^2$ increasing from 0.47 to 0.85 and from 0.15 to 0.8, for stages 4 and 7, respectively. For stage 5, although the geometrically corrected measurements plot closer to the dotted line (where RS2 measurements equal FE predictions), the $R^2$ decreases slightly from 0.72 to 0.67. In these plots, the FE predictions suggest that the horizontal deformation is expected to be relatively slow and therefore, well suited to InSAR (i.e. below the wrapping threshold). Additionally, the fact that the horizontal components of FE and RS2-InSAR compare well suggests that the limitations of InSAR in detecting North–South motion, due to the polar orbit of the SAR satellites, are negligible in the slump area of the TSF. If significant north–south horizontal deformation were present, it would not be captured by the decomposed RS2 east–west component and would therefore not be represented in the projected transverse displacement. Therefore, the comparison presented here may be interpreted as a lower-bound estimate of transverse deformation, consistent with the assumptions of the plane strain FE model.

**Fig. 4:**
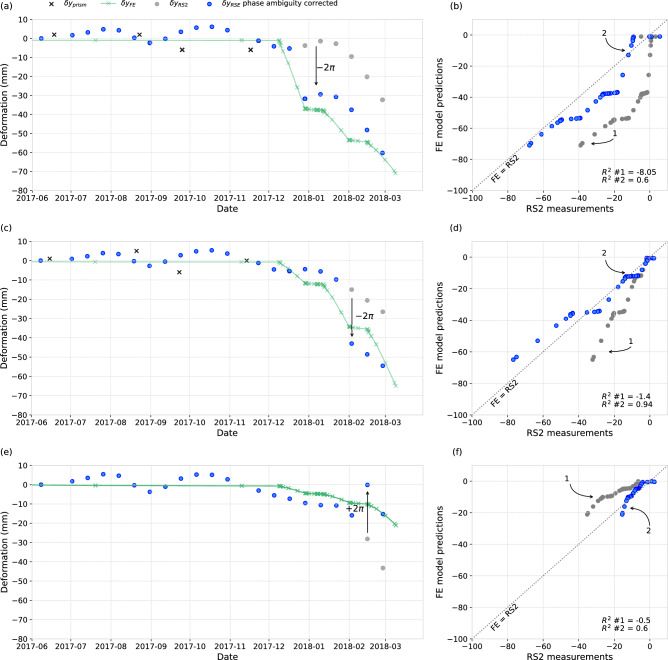
The vertical deformation components ($\delta y$) for prisms, FE, RS2 are plotted. These plots represent the deformation measured at the following stages : (**a**) and (**b**) correspond to stage 4; (**c**) and (**d**) correspond to stage 5; and (**e**) and (**f**) correspond to stage 7. The improvement of the manual phase ambiguity correction of RS2-InSAR is quantified in (**b**), (**d**) and (**f**) (right column); and the dotted line represents the line of perfect fit, where RS2 measurements equal FE model predictions.

On the other hand, the FE predictions suggest that vertical deformation is expected to be rapid during buttress construction, making the InSAR measurements more susceptible to phase ambiguities. The large deviations between $\delta y_{RS2}$ and $\delta y_{FE}$ are illustrated in Fig. 4a–f. The vertical components of RS2-InSAR, $\delta y_{RS2}$, are plotted for the following stages in Fig. 4a,b correspond to stage 4; (c) and (d) stage 5; and, (e) and (f) stage 7. The phase ambiguities are resolved manually following the discussion in the Methods section. Specifically, a single cycle of $2\pi$ is subtracted from the deformation measurements in stages 4 and 5, and $2\pi$ is added in stage 7, as shown in Fig. 4a–c, respectively. The improvements are then quantified in Fig. 4b,d,f (right column). In these figures, the $R^2$ values improved from negative for all stages to 0.6, 0.94 and 0.6, for stages 4, 5 and 7, respectively. Therefore, the phase ambiguity corrected deformations align well with the FE model and confirm that the main differences between FE predictions and InSAR measurements are due to rapid vertical subsidence resulting from buttress construction.

### Integrating high resolution line-of-sight and decomposed InSAR into early warning

**Fig. 5:**
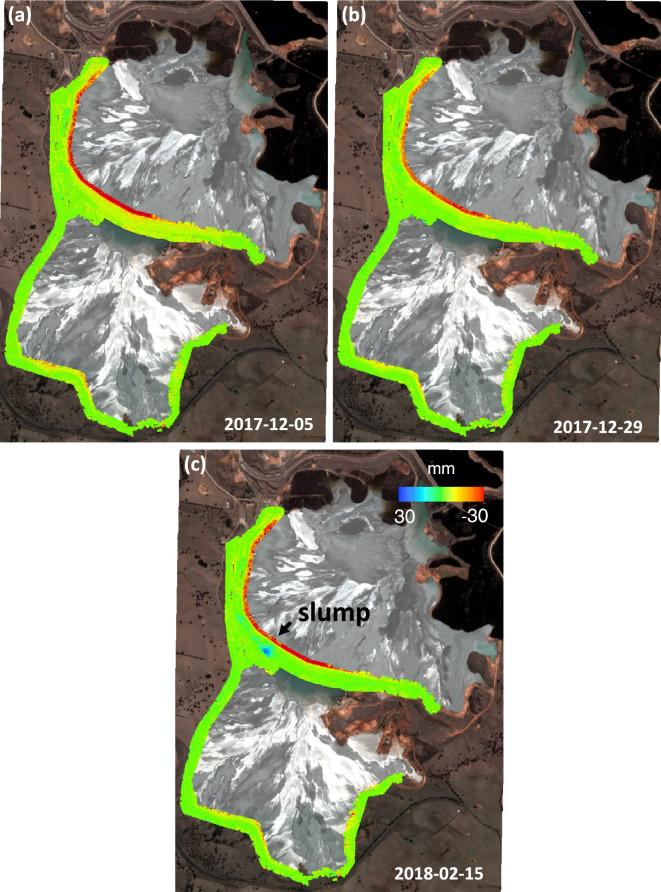
Difference between predicted and measured deformations on different dates on ascending RS-2 InSAR data: (**a**) 2017-12-05, (**b**) 2017-12-29 and (**c**) 2018-02-15. Basemap image copyright 2020 Planet Labs PBC.

**Fig. 6:**
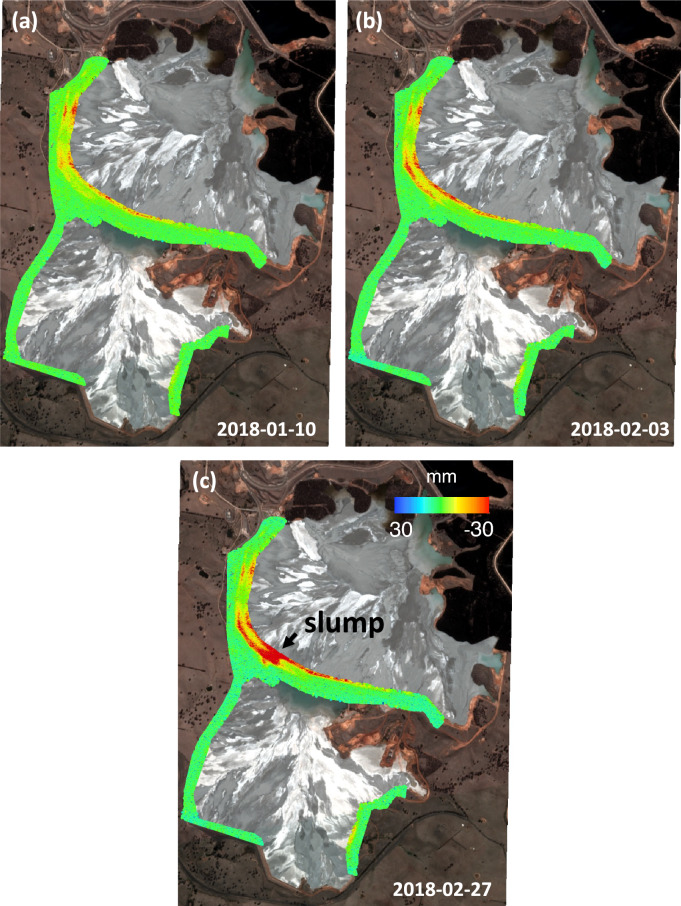
Difference between predicted and measured deformations on different dates on descending RS-2 InSAR data: (**a**) 2018-01-10, (**b**) 2018-02-03 and (**c**) 2018-02-27. Basemap image copyright 2020 Planet Labs PBC.

**Fig. 7:**
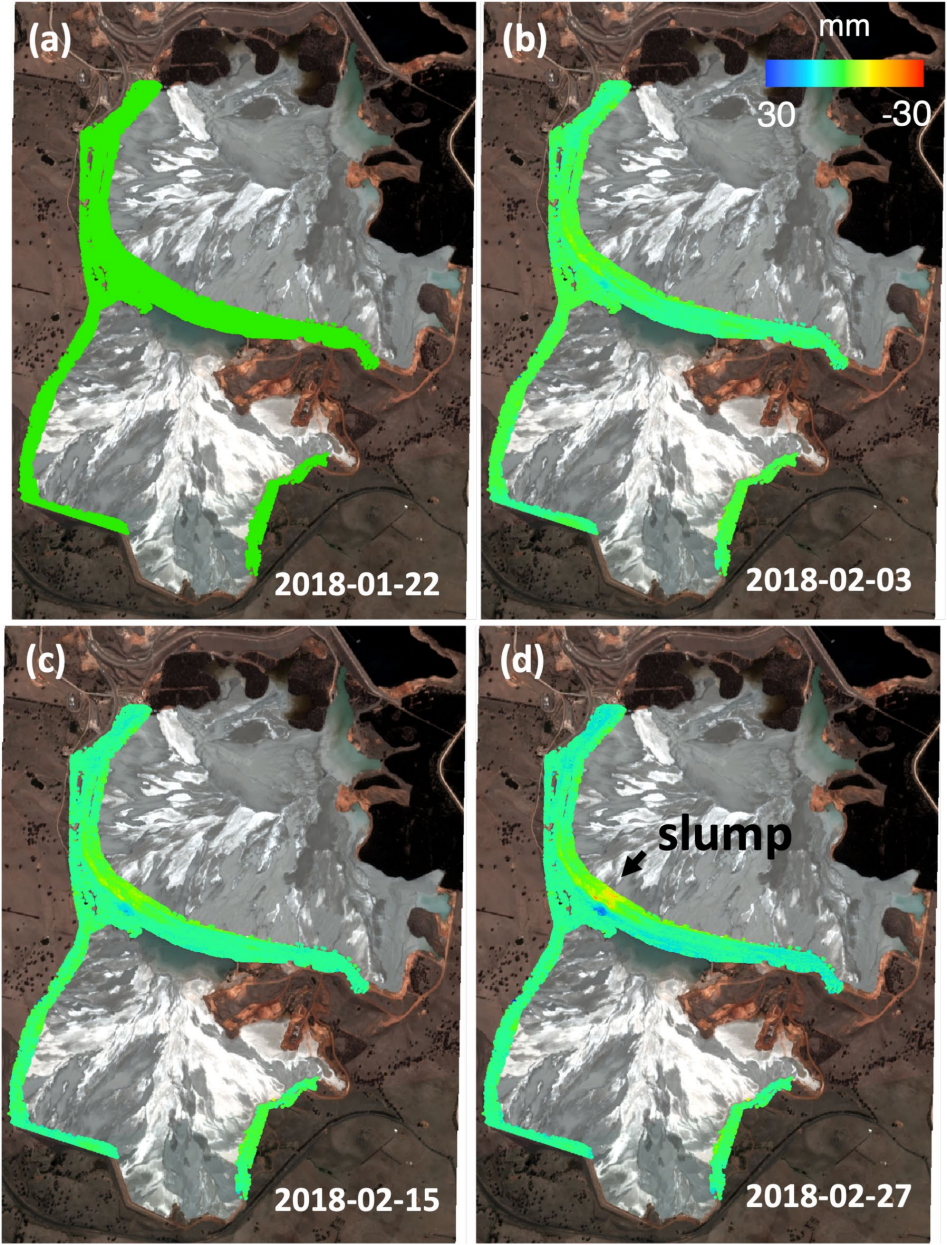
Difference between predicted and measured deformations on different dates for Vertical RS2 InSAR: (**a**) 2018-01-22, (**b**) 2018-02-03, (**c**) 2018-02-15 and (**d**) 2018-02-27. Basemap image copyright 2020 Planet Labs PBC.

**Fig. 8:**
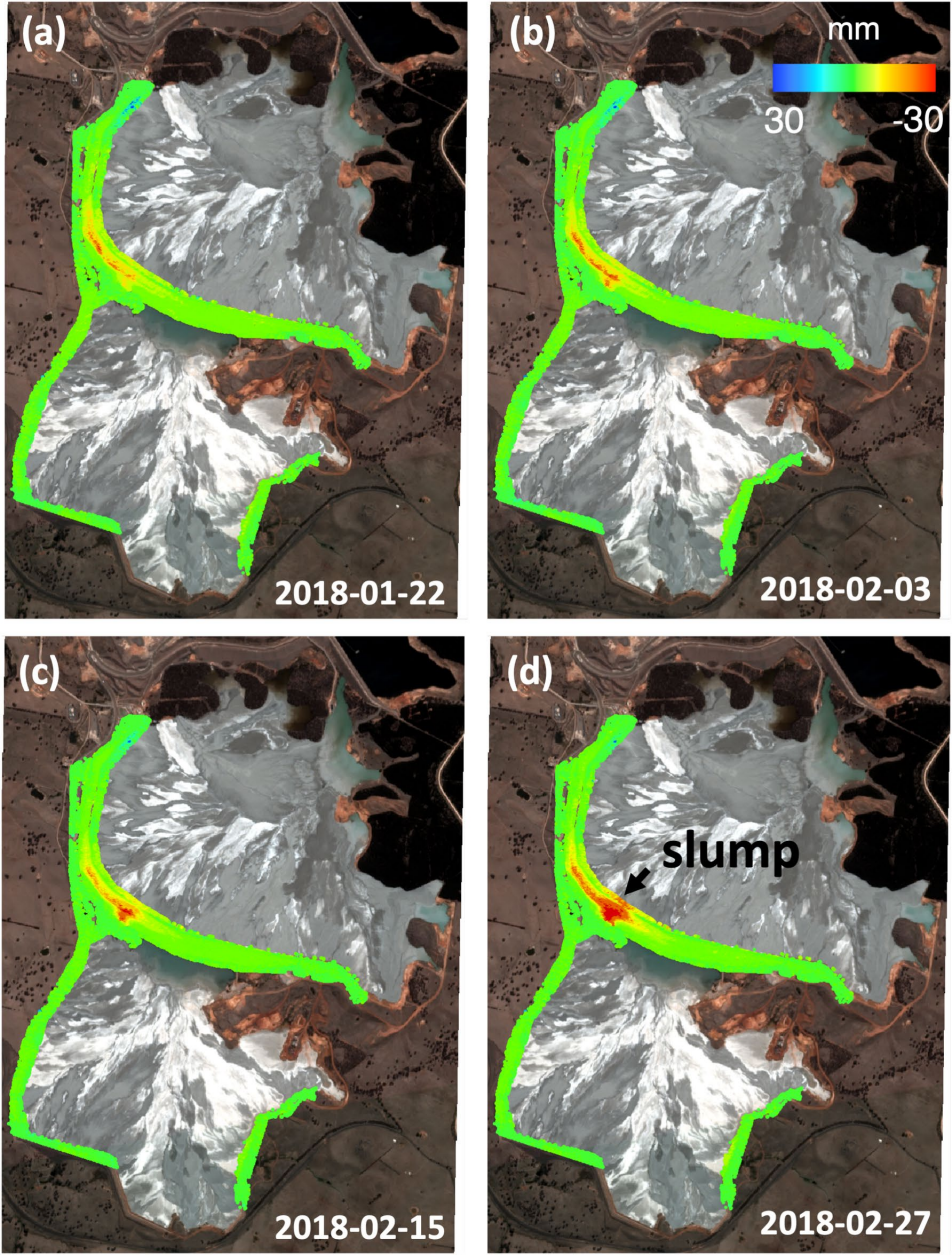
Difference between predicted and measured deformations on different dates for Horizontal RS2 InSAR: (**a**) 2018-01-22, (**b**) 2018-02-03, (**c**) 2018-02-15 and (**d**) 2018-02-27. Basemap image copyright 2020 Planet Labs PBC.

A comparison between machine learning predictions and real measurements are presented in Figs. 5, 6, 7 and 8. In these figures, green areas indicate good agreement between predictions and measurements, resulting in minimal or close to zero differences. In contrast, red and blue areas indicate locations of ’unexpected’ or anomalous deformations. Negative differences (red) indicate locations where the measured deformation is greater than the model prediction in the corresponding InSAR geometry, while positive differences (blue) indicate locations where the measured deformation is smaller than predicted. These figures will be referred to as ’difference maps’ hereon.

The RS2-InSAR ascending and descending difference maps are plotted in Figs. 5 and 6, respectively. In the ascending data, the main rim of the NTSF is red for all the difference maps in Fig. 5 (a)– (c). A noteworthy feature is the cluster of clear anomalous blue clusters visible in the slump area in Figure 5 (c) 2018-02-15. Unfortunately, it is not possible to track the development of this anomalous feature due to the large data gap of around 1.5 months that exists between the ascending dates in Fig. 5 (b) and (c).

Interestingly, the descending RS2-InSAR difference maps in Fig. 6 (a) and (b) reveal the evolution of two red parallel lines over time. These lines become more prominent and merge into a feature reminiscent of the slump morphology in Fig. 6 (c). This observation raises the possibility that anomalous deformation patterns may be tracing the deformation associated with the buttresses. When the two parallel lines merge in Fig. 6 (c), it potentially indicates the ’triggering’ signature of the failure as distinct from buttress construction. Such a clear outline and development of different types of signals are detectable in these figures clearly due to the high spatial resolution of RS2-InSAR and is not visible in S1-InSAR. This potentially implies that it may be possible to distinguish anomalous measurements due to the buttress construction and the triggering of failure. However, given that buttress construction itself contributed to the failure, it is challenging to highlight these signals as being distinctly separate.

Moreover, the lack of clear anomalous signature from the ascending data for Fig. 5 (a) 2017-12-05 and (b) 2017-12-29 may be both due to the satellite orbit geometry or the temporal resolution of the ascending RS2-InSAR. In the descending difference maps, the anomalous deformation due to buttresses in the slump area reveal themselves in Fig. 6 (b) 2018-02-03 which is around one month after Fig. 5 (b) 2017-12-29. Therefore, the reasons for not detecting anomalous deformation in the slump area in Fig. 5 (a) and (b) are probably due to the temporal resolution of the satellite rather than the ascending orbit geometry not being suitable. The fact that both ascending and descending geometries provide clear anomalous clusters in the last acquisition dates may indicate that both geometries are suitable for an early warning of failure for Cadia TSFs.

**Fig. 9:**
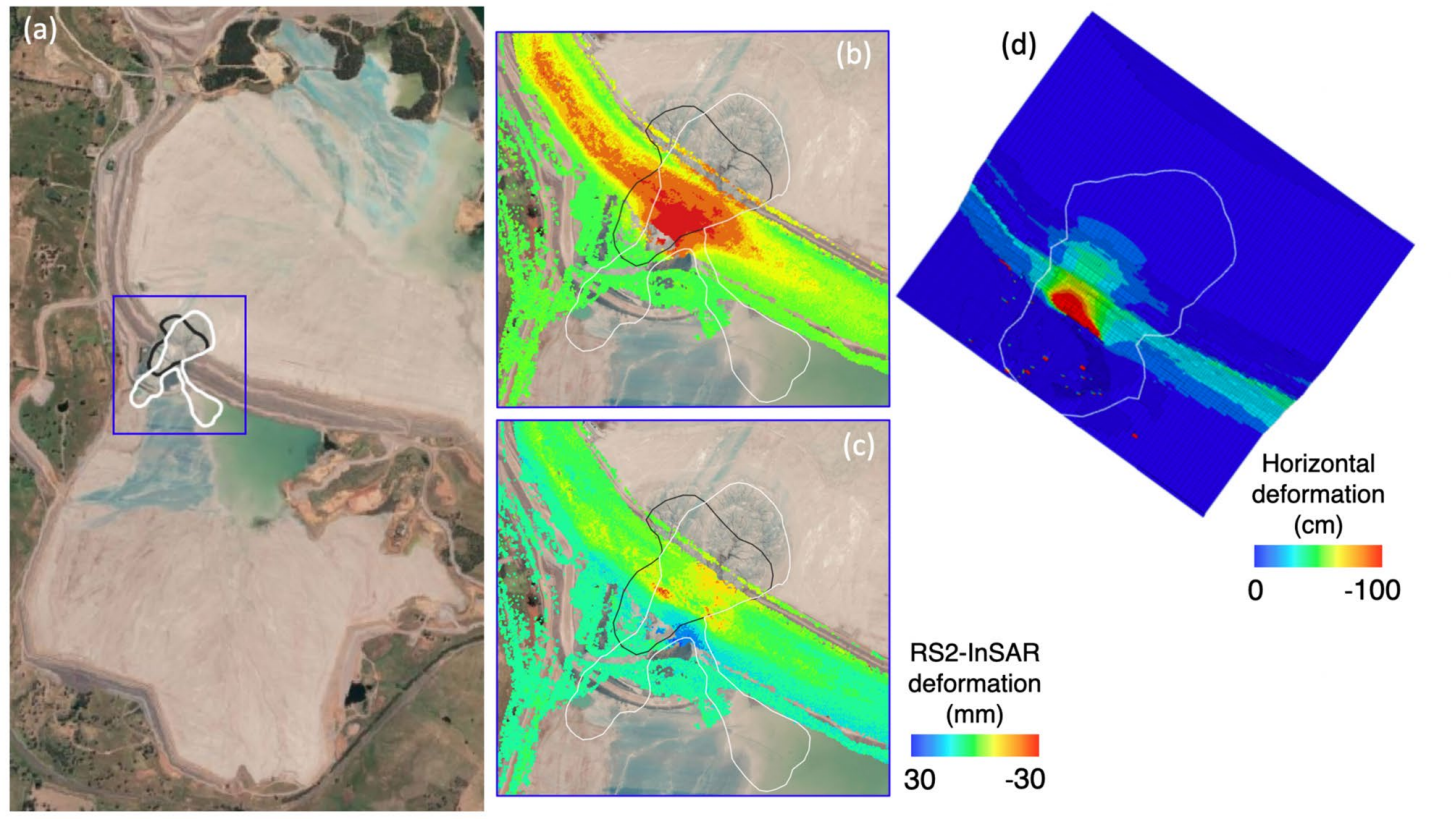
A zoomed in view of the failure area. (**a**) Location and extent of failure, which occurred in two stages, as outlined in white and black polygons (black predates white). The difference between predicted and measured deformations on 2018-02-27 RS2 InSAR (**b**) horizontal and (**c**) vertical. (**d**) The horizontal displacement contours from 3D numerical modelling results for 2018-03-08, from Jefferies et al. (2019)^7^.

The decomposed RS2 vertical and horizontal difference maps are illustrated in Figs. 7 and 8, respectively. The evolution of the vertical anomaly is made up of positive (blue) and negative (red) deformation clusters adjacent to each other, as seen in Fig. 7 (b) – (d). This implies the presence of two different deformation mechanisms, resulting in the EE-DL model to either underestimate (red) or overestimate (blue) the measurements. A zoomed-in view of the failure area and a further interpretation of these clusters are available in Fig. 9 (c). The anomalies in the slump area are most evident in the RS2 horizontal difference maps visualised in Fig. 8 (a) – (d). The slump area is outlined as a distinct cluster adjacent to the anomalous deformations parallel to the dam. The slump area outline can be visually identified as early as 2018-01-22. This is the earliest point at which the anomaly can be observed compared to the other RS2 and S1 InSAR datasets. It makes sense that the horizontal motion components are best able to capture the failure signature, because horizontal motion is more directly related to failure than vertical motion.

The most evident difference between the decomposed RS2 difference maps (Figs. 7 and 8) and the LOS difference maps (Figs. 6 and 5) is the reduction or disappearance of the large red features along the northern TSF dam rim. In the LOS difference maps, linear anomaly patterns are present that are not uniquely associated with the failure area. This does not imply that LOS anomalies are erroneous; rather, they may include geometry-dependent components that are less informative for failure-related interpretation. When ascending and descending observations are combined and decomposed into vertical and horizontal components, these geometry-specific signals are largely suppressed. The remaining anomalies in the decomposed difference maps are spatially coherent with the known failure area, indicating that the performance of the deep learning model trained on decomposed deformation components improve compared to LOS data.

Additionally, the anomalies observed in the the horizontal and vertical difference maps are zoomed-in and discussed within the context of potential geotechnical interpretation in Fig. 9 (a) – (d). The location and extent of the TSF failed area are depicted in Fig. 9 (a). Figure 9 (b) and (d) provide a zoomed-in view of the failure area within the horizontal and vertical difference maps presented in Figs. 7 (d) and 8 (d). The highly anomalous regions (red) in the horizontal difference maps match with key observations, including the location of the failure area and the footprint of modelled horizontal deformations across the slump width in Fig. 9 (d), which are the horizontal deformations predicted by 3D numerical analysis from Jefferies et al. (2019)^7^.

**Fig. 10:**
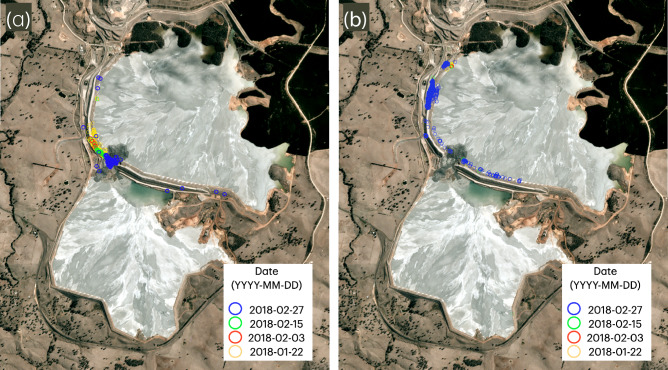
RS2 InSAR horizontal anomalies, which are defined as points lying outside the 95% confidence data ellipse fitted to the predicted–measured deformation scatter. Anomalies above and below the confidence bounds are shown in panels (**a**) and (**b**), respectively. The thickness of the circles indicates the coincidence of multiple measurements at the same locations over the analysed dates. Basemap image copyright 2025 Planet Labs PLC.

Finally, the anomalous measurements in the RS2-InSAR horizontal difference maps are shown in Fig. 10 (a) and (b). These anomalous measurements are identified using the joint distribution of predicted and measured deformation. All measurements lying outside the 95% confidence data ellipse in predicted–measured space are classified as anomalous for the acquisition dates 2018-01-22, 2018-02-03, 2018-02-15, and 2018-02-27. The thickness of the plotted circles indicates repeated anomalous detections at the same spatial locations across multiple dates. Notably, the anomalies in Fig. 10 (a) coincide with the slump area, whereas the anomalies in Fig. 10 (b) appear to capture other types of movement, potentially related to upstream tailings behaviour or buttress construction activities. This approach demonstrates how anomalies identified through the early warning analysis can be further classified according to their distinct deformation behaviours.

## Conclusion and discussion

An opportunity for novel cross-technology calibration is demonstrated through the use of FE predictions to correct for phase ambiguities in PS-InSAR data. This calibration strategy suggests that PS-InSAR is not sufficient on its own for rapid deformation behaviour prediction. The same consideration cannot be done for ISBAS, because it does not display clear indications of phase unwrapping errors. This is likely due to the nature of SBAS-type methods, which includes ’smoothing’ (or ’multi-looking’) of the signals. Such averaging of the signals across pixels likely dampens these high magnitude irregularities. Therefore, it is challenging to correct for issues that cannot be detected in the first place.

The advantages of ISBAS over PS-InSAR lie in its ability to maintain a high spatial density of measurements even when processed over long periods of time. In contrast, applying PS-InSAR for extended periods is challenging because measurement coherence is easily lost. Despite the potential underestimation of the deformation, ISBAS still successfully detects spatially anomalous patterns over the slump area^25^. Therefore, the spatial distribution of anomalous deformation are better captured in ISBAS compared to PS-InSAR despite the phase ambiguities. Several InSAR processing approaches aim to combine the advantages of both PS-InSAR and SBAS, leveraging the strengths of both methodologies. However, in this study, maintaining separate control over the two processing methods and analysing them independently enabled a more thorough exploration of their respective advantages and limitations. Overall, PS-InSAR and ISBAS should be viewed as complementary techniques for TSF monitoring, as each provides distinct insights into the TSF behaviour. While ISBAS may be more suitable than PS-InSAR for long term monitoring of TSFs, the aforementioned limitations suggest that a more reliable system should also include PS-InSAR and FE modelling.

Both InSAR and geotechnical modelling are subject to inherent uncertainties; however, when combined, they offer tools to probe and reduce the uncertainties present within each method. The phase ambiguity corrected S1 PS-InSAR data shows strong agreement with both high resolution RS2 InSAR and FE predictions in terms of deformation magnitude and trend for TSF stages 4 and 5. This suggests that the limitations of S1-ISBAS are not inherent to the InSAR method nor the characteristics of the S1 satellite. By re-processing S1 with PS-InSAR and correcting for its phase ambiguities guided by FE predictions, it is possible to capture the rapid deformation behaviour resulting from the construction of buttresses.

Interestingly, for TSF stage 7, there is strong agreement between the S1 PS-InSAR ambiguity-corrected data and RS2-InSAR measurements, but not with the FE model. The fact that two independent InSAR methods and SAR data agree is significant, but it is challenging to resolve these discrepancies without an external ground truth data. The prisms cannot be employed for this, because they were removed during this time period to accommodate construction activities. However, the low sensitivity of InSAR to the North - South direction can be ruled out, since the horizontal components of RS2-InSAR and FE predictions align well. Therefore, it is likely due to known SAR signal distortions, such as layover and foreshadow, resulting from the steep geometry of the higher TSF stages and the small spatial footprint of stage 7.

Moreover, if the InSAR datasets represent the truth, then it highlights the limitations of the FE model in accurately capturing the deformation of higher dam stages. This is potentially due to simplifications and uncertainties in the model parameters, such as the tailings properties. The deformation signals of higher dam stages such as stage 7 are dominated by the deformation of the tailings^25^. And within the context of a remote early warning system, such discrepancies between measurements and models should trigger an alert for further detailed in-situ analysis to resolve it.

One of the advantages of EE-DL is its flexibility and ease with which other types of data can be integrated into the workflow. To demonstrate this, a variety of InSAR data types with different resolutions and measurement types were incorporated into the EE-DL workflow. The transition from S1 to RS2 data represents an order of magnitude increase in data volume. To accommodate this scaling, several adaptations were required, including changes to the embedding creation rule and re-tuning of hyper-parameters. Additionally, both line-of-sight and decomposed motion components of RS2 InSAR measurements were successfully incorporated into EE-DL.

The early warning capabilities performed best with the horizontal motion component of the RS2 InSAR data. As expected, the EE-DL predictions based on RS2 InSAR data provided more detailed insights into the development of anomalous deformation patterns than S1, likely owing to its higher spatial resolution. As expected, RS2 horizontal deformation data led the best early warning capabilities. It is the only data in which the failure area can be observed as early as 2018-01-22, with distinct clusters sticking out of the mass of deformation parallel to the TSF. Although, a similar outline can be visually identified in the vertical results, it is not visually clear until 2018-02-27. This clarity of the failure signature in the horizontal data makes sense, as horizontal motion (i.e. strain) is more directly related to failure than vertical motion.

Both ascending and descending orbit datasets are suitable for early warning of failure at Cadia TSF. Both descending and ascending data detect visually clear anomalies at their last temporal acquisitions, which correspond to 2018-02-27 and 2018-02-15, respectively. Due to the differing temporal resolutions and the fact that they are acquired at different dates, it is challenging to compare them fairly and determine which dataset performs best. Although this may indicate that both orbital geometries are suitable for detecting the slump at Cadia TSF, it does not rule out the possibility that one geometry may end up being more suitable than the other, especially if the slump occurred elsewhere on the TSF. As expected, the development of the anomalies are best observed from the decomposed motions, rather than either the ascending or the descending datasets. This improvement is likely due to the higher temporal resolution of the decomposed datasets, which represent the combined insights of both acquisition geometries.

The proposed InSAR–FE–DL framework is compatible with practical deployment in active mining environments, as it leverages routinely acquired satellite data and does not require continuous in-situ instrumentation. Monitoring frequency is primarily governed by satellite revisit intervals, which for contemporary SAR missions typically range from 6 to 12 days for open-access sensors and can be higher for commercial constellations. The framework is therefore well suited to near-real-time monitoring, where updates are performed at each new acquisition rather than continuously. The FE modelling does not need to be updated at every acquisition; instead, FE predictions can be refreshed episodically following major construction activities or when persistent discrepancies are detected. In this context, the framework supports periodic reassessment rather than continuous recalibration, making it feasible for integration into existing mine monitoring workflows as a decision-support and early-warning tool.

There is value in EE-DL beyond early warning, particularly in data discovery. Interpreting the spatial distribution of InSAR-derived deformations over TSFs is challenging, as deformation signals of varying magnitudes often coexist due to ongoing consolidation. Therefore, EE-DL is demonstrated as a potential tool to help focus on areas of unexpected deformations, aiding in making sense of the large collection of measurements. For example, RS2-InSAR descending data within EE-DL revealed the development of unexpected deformation patterns that appear to correspond to buttress construction. The EE-DL results can then be further validated using other types of satellite data, including SAR back-scatter and multi-spectral optical data. The large differences in the stress states between areas with and without buttress cover can then be evaluated with the FE model. This is a critical aspect of the digital twin vision, as it allows the capturing of the dynamic state of the TSF by calculating metrics such as the degree of completion of buttresses construction. By integrating insights from satellite remote sensing, geotechnical modelling and deep learning, this study has addresses some of the most fundamental research components that are currently missing, before satellite enabled digital twin systems may be achieved for the monitoring of TSFs.

Finally, it is important to note that both cost and data availability factors are changing rapidly. With the current trend of increasingly cheaper launch capabilities and instrument developments, the cost of commercial satellite data is likely to keep reducing and therefore, the availability of high resolution data is likely to keep increasing^46^. In parallel, the financial cost of recent TSF failures in terms of their impact on stock price, social compensation and environmental remediation are in the range of USD 750M to USD 56B. These costs are significantly lower than the estimated costs of hypothetical, highest specification real-time monitoring technologies, which are estimated in the range of USD 400k to USD 500k^47^. Therefore, there is a critical need and an economically defend-able case for a global monitoring capability for TSFs to protect local communities, surrounding natural habitats and ecosystems from toxic mine waste.

## Methods

### Cadia TSF case history

**Fig. 11:**
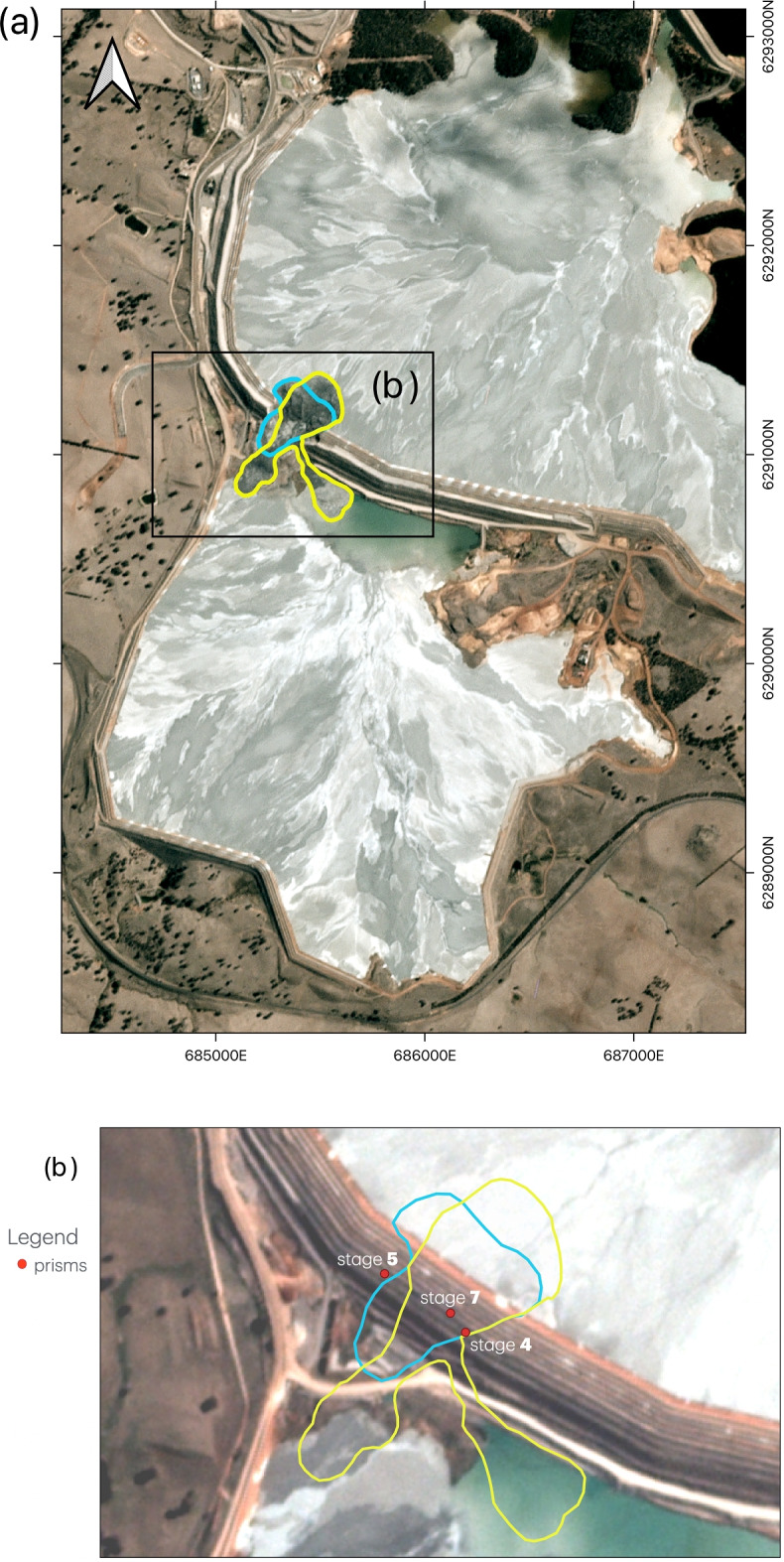
The geographical setting of the Cadia TSFs and the failure event. (**a**) The Cadia failure developed in two stages, as outlined in blue and yellow (blue predating yellow). The data coordinates are in WGS 84 / UTM zone 55 South with EPSG : 32755. The basemap image was acquired after the failure, on 16-03-2018 (DD-MM-YYYY) Basemap image copyright Planet Labs. (**b**) The basemap image depicts the Cadia TSF before the failure, on 30-09-2017 (DD-MM-YYYY). The approximate location of the three prisms available within the failure area are shown in red. Basemap image copyright Planet Labs.

The Cadia mining complex in south-western Australia comprises two tailings storage facilities, located to the north and south of the site. The failure occurred at the northern TSF on 9 March 2018, as annotated in Fig. 11 (a). Figure 11 (a) illustrates the spatial footprint of the failure, which developed in two stages (blue predating yellow). A zoomed-in view of the failure area, overlaid on a pre-failure basemap, is shown in Fig. 11 (b). Figure 11 (b) also indicates the locations of available ground-based deformation measurements derived from prism monitoring.

The failure was attributed to the presence of a brittle, low-density foundation soil layer known as Forest Reef Volcanics (FRV) Unit A. This unit is a highly weathered layer of the bedrock, which was not identified prior to the failure and was only discovered through the post-failure investigation conducted by Jefferies et al. (2019)^7^. The post-failure investigation provides an unprecedented level of public access to fundamental geotechnical datasets, including dam geometry, construction sequences and raw laboratory test data characterising the dam and foundation materials at the Cadia TSF. Such high-quality ground-truth data and associated insights are rarely publicly available for TSF failures.

**Fig. 12:**
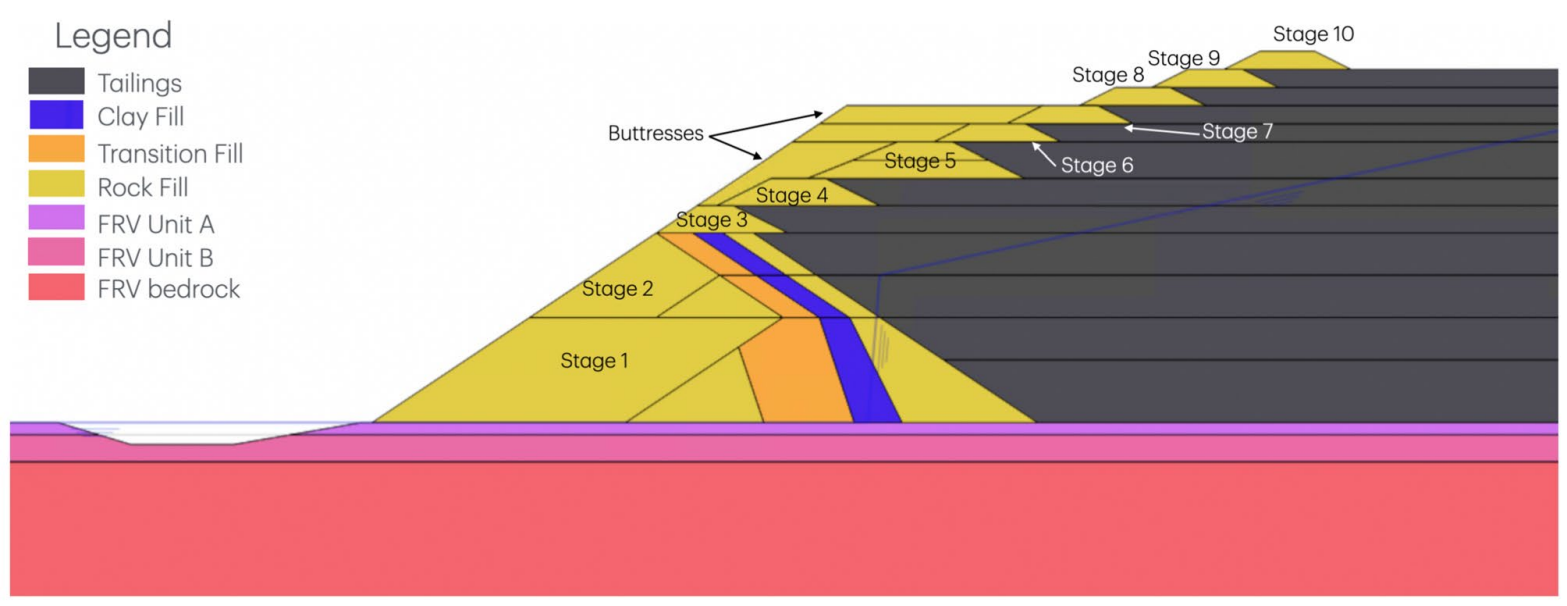
The configuration of the Cadia northern Tailings Storage Facility that experienced a failure. The stages correspond to successive phases of dam construction. Detailed geotechnical laboratory test results and adopted soil constitutive models are presented in Jefferies et al. (2019)^7^ and Bayaraa et al. (2024)^25^.

The geometric configuration of the Cadia TSF that experienced failure is shown in Fig. 12, which presents a schematic cross-section through the failure zone. The stages shown correspond to successive phases of dam construction and indicate that most of the facility was constructed using the upstream method, with failure occurring in stages 4 through 10. In contrast, the lower stages (stages 1 to 3) are founded directly on natural ground or earlier dam structures, whereas the upstream stages are predominantly founded on previously deposited tailings. In principle, upstream raises are constructed on consolidated tailings; however, ensuring full consolidation of all underlying tailings layers is often challenging, thereby introducing additional geotechnical risk. The re-interpretation of soil parameters and the development of the finite-element constitutive soil model adopted in this study are described in Bayaraa et al. (2024)^25^. Given the scale of the dam, plane-strain conditions are assumed. Coupled consolidation analyses are employed to reproduce the timing of dam construction and tailings deposition stages, as well as the time-dependent behaviour of the dam and foundation materials, using the finite-element package PLAXIS 2D (2019).

### InSAR data and processing

’Sentinel-1’ is a C-band SAR satellite from the European Space Agency’s Copernicus program. Relative to high-resolution commercial satellites, S1 data are routinely operated in Interferometric Wide Swath mode using the Terrain Observation with Progressive Scans (TOPS) technique, which provide an intermediate spatial resolution of 5 m in range and 20 m in azimuth at a temporal resolution of 6 or 12 day revisit depending on the location on Earth^48^. In the case of the Cadia failure, RS2 data is the only commercial InSAR data available for the period preceding the failure. RS2 is also a C-band SAR satellite and is available at Spotlight mode, which provides the highest spatial resolution, approximately 1.3 m in azimuth and 3.9 m in range. Such high spatial resolution is not globally guaranteed, as RS2 supports multiple acquisition modes that result in coarser spatial resolutions; some of these modes offer spatial resolutions that are comparable to or even coarser than those of S1^49^. Although, S1 also supports other acquisition modes, such as Stripmap, which provide finer spatial resolutions of approximately 5 m × 5 m; however, these modes are not routinely acquired globally like the TOPS mode. The InSAR data sources are described in Tables 1 and 2 for RS2 and S1, respectively. The RS2-InSAR datasets are composed of data from both the line-of-sight (LOS) ascending and descending orbits, as well as the decomposed motions. Table 1 shows that the frequency for descending data are stable at 24 days. However, the frequency for the ascending stack is mostly 24 days, with two acquisition gaps that result in 48 days. In comparison, the decomposed vertical and horizontal components are at a frequency of 12 days, with one data gap resulting in 24 days.

**Table 1:** Radarsat-2 InSAR data description. Descending LOS data frequency is stable, whilst ascending LOS data frequency is mostly 24 days with two acquisition gaps that result in 48 days. The vertical and horizontal components are at mostly 12 day frequency, with one data gap resulting in 24 days.

InSAR deformation	Date	(YYYY-MM-DD)	Acquisition
	From	To	Frequency (days)
Descending LOS	2017-06-08	2018-02-27	24
Ascending LOS	2017-07-14	2017-10-18	24
	2017-10-18	2017-12-05	48
	2017-12-05	2017-12-29	24
	2017-12-29	2018-02-15	48
Vertical and horizontal	2017-06-08	2017-07-02	24
	2017-07-02	2018-02-27	12

**Table 2:** Sentinel-1 InSAR data processed with ISBAS and PS-InSAR algorithms. Sentinel-1 data over Cadia TSF is available from the descending orbit only.

InSAR deformation	Date	(YYYY-MM-DD)	Acquisition
	From	To	Frequency (days)
ISBAS	2015-12-02	2018-02-25	12
PS-InSAR	2016-12-08	2018-02-25	12

The impact of the InSAR processing algorithm is investigated by processing the S-1 data using a variant of the SBAS algorithm^50^, called intermittent-SBAS (ISBAS)^51^ and Persistent Scatterer Interferometry (PS-InSAR)^52^. Since S1 data over Cadia are available only from a descending stack, it is not possible to decompose the line-of-sight deformation into its vertical and horizontal components. Therefore, S1-InSAR is compared to the equivalent RS2-InSAR data from descending orbit only. Additionally, these measurements are compared to available geotechnical simulations and ground based prisms.

Due to licensing agreements between satellite operators and various governments, direct access to raw RS2 data over Cadia is restricted. However, this study was granted access to derived InSAR deformation products, as described by Hudson et al. (2021)^24^, courtesy of MDA Space Ltd. Consequently, this restriction limits the ability to assess the impact of alternative processing approaches on RS2 data.

Table 2 shows that the ISBAS processing includes a SAR data stack spanning over 2 years, while PS-InSAR processing has been applied to a shorter stack, covering a total of just over 1 year. PS-InSAR requires re-processing of S1 data with a shorter temporal stack, to increase the likelihood of obtaining measurements. This is due to the scattering behaviour that may change over time, reducing the probability of finding persistent scatterers with consistent behaviour over extended periods. Moreover, PS-InSAR was applied to the S1 data stack by the authors using the ENVI SARScape software package (NV5 Geospatial), following established PS-InSAR processing methodologies^52^. PS-InSAR may help resolve the discrepancies observed between FE model and ISBAS immediately preceding failure observed in Bayaraa et al. (2024)^25^. ISBAS is suited for distributed scatterers, where the deformation signal is uniform within a larger area, as the resolution cells are ’multi-looked’, e.g. interpolated to a lower resolution. In contrast, PS detects strong scatterers in each pixel and therefore, the deformation signal is dominated by the movements of the strong scatterers, and no multi-looking might be performed.

**Fig. 13:**
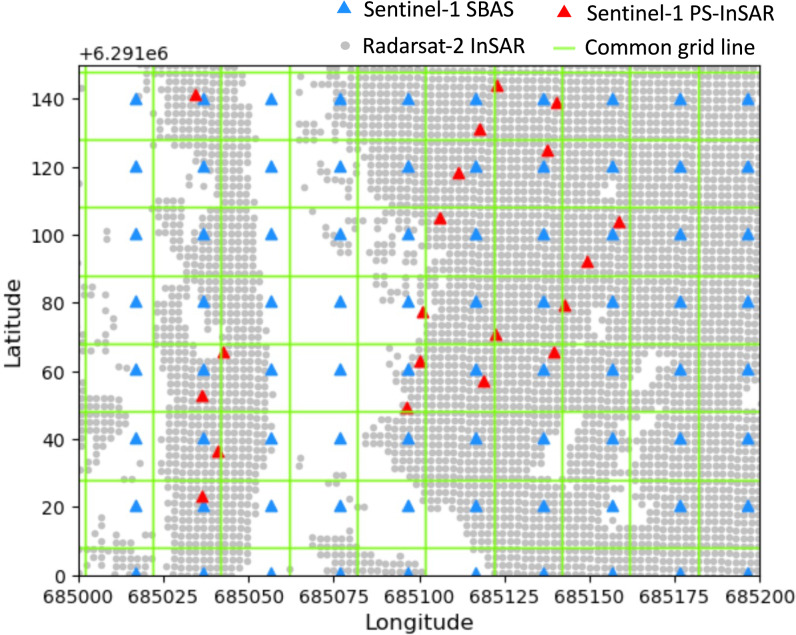
Visualisation of the spatial resolution of Sentinel-1 SBAS, PS-InSAR and Radarsat-2 InSAR data, overlaid with a common 20m $\times$ 20m grid of pixels.

The S1 and RS2-InSAR data are acquired at different spatial and temporal resolutions, and therefore require pre-processing steps before they can be compared, such as the approaches described in Sadeghi et al. (2021)^53^. To enable a spatial comparison of S1 and RS2-InSAR measurements, firstly, a common grid of pixels is defined with a size of 20m $\times$ 20m. To highlight the differences in spatial resolution and distribution of InSAR measurements, S1-based ISBAS and PS-InSAR are plotted alongside the common grid and the RS2-InSAR measurements in Fig. 13.

### Deformation alignment

The deformation predictions from the FE model and the prism measurements are re-projected into the line-of-sight (LOS) of the Sentinel-1 SAR satellite to enable comparison with the InSAR measurements. This is achieved by combining the vertical and horizontal deformation components and transforming them into LOS using the relationship developed in Selvakumaran et al. (2020)^54^:

1 $$\begin{aligned} \begin{aligned} \delta _{LOS}&= \begin{pmatrix} \cos \theta \\ \sin \theta \cos \alpha \\ \sin \theta \sin \alpha \end{pmatrix} * \begin{pmatrix} \delta _{v} \\ \delta _{L} \\ \delta _{T} \end{pmatrix} \\ \end{aligned} \end{aligned}$$

**Fig. 14:**
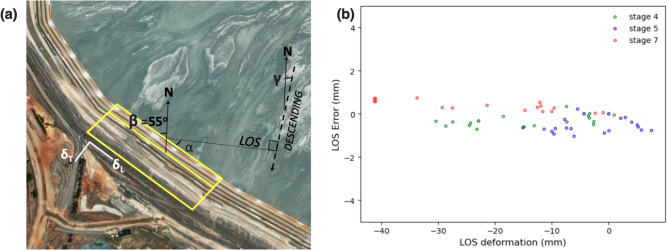
(**a**) Illustration of InSAR LOS deformations and the geometrical relationship between the TSF orientation and the descending satellite flight path. Basemap image copyright 1995–2020 Esri. (**b**) Despite differences in sensor configurations of descending RS2 and S1, the resulting LOS measurements are highly comparable, with only negligible differences. Example prism measurements are converted into LOS and the resulting differences between the RS2 and S1 configurations are plotted as LOS Error.

Here, $\theta$ denotes the sensor incidence angle, defined as the angle between the SAR signal path and the vertical. The geometrical relationships between the satellite LOS and the dam orientation are illustrated in Fig. 14a, with the parameters summarised in Table 3. The geometry of the failed TSF section is defined by $\beta$ = 55$^o$. The dam segment orientation relative to the LOS is captured through $\alpha$. While, $\gamma$ represents the satellite heading angle, which is measured clockwise from the North, equivalent to 180$^o$ minus the nominal scene heading angle. The vertical component of the displacement is $\delta _{v}$ and the horizontal component is further resolved in the direction longitudinal $\delta _{L}$ and transverse $\delta _{T}$ to the dam axis. Moreover, Fig. 14b presents the LOS Error, defined as the difference between prism measurements projected using RS2 and S1 descending sensor geometries. The results confirm that the LOS deformation measurements derived from the two descending configurations are comparable, with only negligible differences.

**Table 3:** Configurations of descending Sentinel-1 and Radarsat-2 satellites.

Sensor	Scene heading angle	$\alpha$	$\theta$
Sentinel-1	167$^{\circ }$	22$^{\circ }$	35.5$^{\circ }$
Radarsat-2	163$^{\circ }$	18$^{\circ }$	38.5$^{\circ }$

**Fig. 15:**
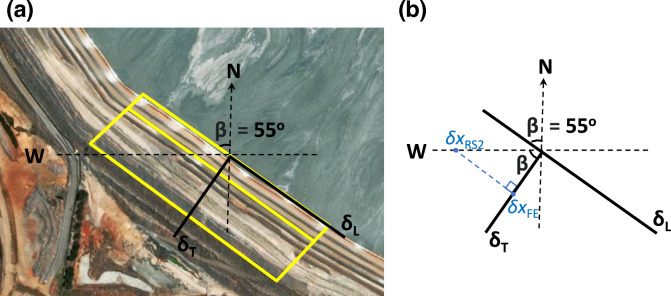
(**a**) Diagram for mapping RS2-InSAR horizontal motion ($\delta x_{RS2}$) onto FE horizontal deformation ($\delta x_{FE}$). FE horizontal motion is in the direction transverse to TSF, whereas the horizontal motion derived from InSAR is in the East to West direction. Basemap image copyright 1995-2020 Esri. (**b**) Trigonometric transformation of $\delta x_{FE}$ onto $\delta x_{RS2}$.

Moreover, to enable comparison of the decomposed RS2-InSAR motions to results from geomechanical FE model and prism measurements, the horizontal motion components are geometrically aligned, as each measures deformation along different directions. The horizontal motion derived from RS2 InSAR ($\delta x_{RS2}$), defined in the geographic east–west direction, cannot be directly compared to the transverse deformation predicted by the FE model ($\delta x_{FE}$). Therefore, $\delta x_{RS2}$ is projected onto the FE transverse direction using the local dam orientation ($\beta$ = 55$^o$ from North), assuming negligible longitudinal deformation ($\delta _{L}$) under plane-strain conditions, as illustrated in Figures 15 (a) and (b). The horizontal component of the motion derived from RS2 is plotted as $\delta x_{RS2}$ in the Westward direction defined by ’W’ and from FE is plotted as $\delta x_{FE}$ in Fig. 15 (b). The FE model represents the slump area, which is located in a TSF section of $\beta = 55^\circ$ from the North. The $\delta x_{RS2}$ can be projected onto $\delta x_{FE}$ following Eq. 2, which represents a vector projection of the RS2-derived horizontal displacement onto the FE transverse direction and does not attempt to reconstruct the full horizontal displacement magnitude.

2 $$\begin{aligned} \delta x_{FE} = \delta x_{RS2} cos(\beta ) \end{aligned}$$

### Phase unwrapping correction

**Fig. 16:**
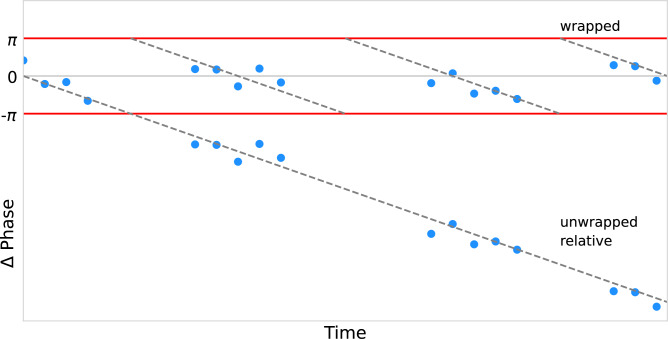
A simple example of wrapped and unwrapped phases. The wrapped signal is modulo $2\pi$ (1 cycle) of the relative unwrapped signal. Modified from^59,60^.

The basic principles of phase unwrapping are used to demonstrate the extent to which phase ambiguity and uwrapping errors may explain the large differences observed between ISBAS and FE predictions following buttress construction at Cadia in Bayaraa et al. (2024)^25^. Once interferograms are generated as part of InSAR processing, the deformation components of the phase are separated from unwanted components such as noise. Because SAR Interferometry measures the relative phases between acquisitions, these steps result in phase measurements observed in cycles of $2\pi$ for each SAR acquisition, also referred to as ’wrapped phases’, as illustrated in Fig. 16. The figure demonstrates the basics of wrapped phase measurements being ’unwrapped’ through a linear deformation model. According to Itoh’s condition, the difference in phases between two adjacent samples cannot exceed $\pi$, ensuring that the phase values lie within the range of $-\pi< phase < \pi$^41^. This means, firstly, the difference in phase between two adjacent samples must be calculated. If the difference is more than $\pi$, then a cycle (2 $\pi$) is subtracted. In contrast, if it is less than $-\pi$, then a cycle is added. The number of cycles of 2 $\pi$ that needs to be added or subtracted is unknown and depends on the particular phase unwrapping methodology and assumptions.

The simplified phase unwrapping strategy described in Fig. 16 follows a linear deformation model, which is commonly assumed in PS-InSAR approaches^52^. Alternative motion models, including non-linear and seasonal deformation have also been proposed in the literature to capture more complex behaviour^55,56^. The linear model is retained in this study as a pragmatic and widely used assumption, that enables phase ambiguity effects to be isolated and examined within the context of physics based FE-guided interpretation.

Phase unwrapping is a critical research area, containing a variety of approaches to the problem^57,58^. Figure 16 illustrates a one-dimensional phase unwrapping example, which is based on the signal’s behaviour through time. Other methods involve phase unwrapping in two spatial dimensions. However, as the number of dimensions increase, additional challenges emerge, such as path dependency. The specific starting location, the direction and the path followed during the phase unwrapping process can lead to different solutions. This is also called the ’residue theory’, where the location of the residues then inform the ’jump’ in the phases. The details and the complications of advanced phase unwrapping techniques are beyond the scope of this study.

### Early warning methodology

Early warning systems aim to detect abnormal or anomalous deformation before failure occurs. The Embedded Entities within Deep Learning (EE-DL) framework^36^ was developed to accommodate inherent characteristics of the InSAR time-series data that limit more conventional deep learning approaches. InSAR measurements are spatially sparse, irregularly distributed, temporally asynchronous, and are accompanied by high-cardinality categorical metadata such as acquisition geometry, spatial location and time indices. EE-DL enables these heterogeneous inputs to be represented efficiently through learned embeddings, without requiring spatial gridding, image interpolation, or regular temporal sampling. In contrast to convolutional or recurrent architectures, which typically assume dense spatial fields or uniform time steps, EE-DL preserves the native structure of InSAR observations while scaling efficiently to large datasets. This is particularly important for tailings storage facilities, where deformation signals coexist with background consolidation and spatial data gaps are common. The objective of the deep learning component in this study is not to perform deterministic failure prediction, but to identify statistically anomalous deformation patterns relative to learned baseline behaviour. Within this context, EE-DL provides a flexible and computationally efficient framework for early-warning and data-discovery tasks. Previous work has demonstrated the effectiveness of this approach through quantitative comparison with more conventional and explainable machine-learning models, including the common baseline model random forest and probabilistic Gaussian process regression, where EE-DL showed improved performance in capturing the spatio-temporal deformation patterns in S1 InSAR data (Bayaraa et al. 2023).

Since the objective is to evaluate the performance of the EE-DL model on different types of InSAR datasets, each dataset is analysed independently. Apart from the decomposed vertical and horizontal components of RS2-InSAR, no dataset merging has been performed.

**Fig. 17:**
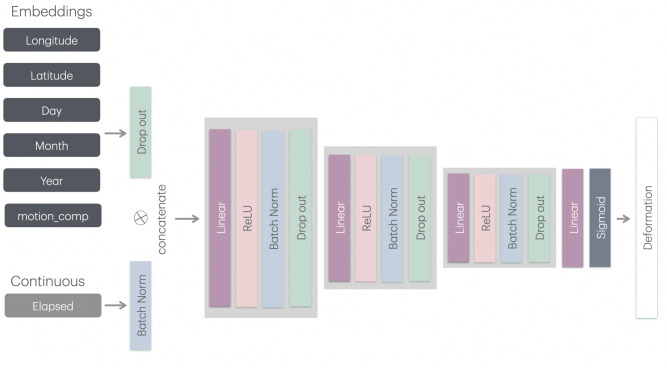
Embedded-entities within the deep learning framework. The model architecture is a fully connected, three-layer neural network with hidden layers comprising 1000, 500 and 250 neurons, respectively.

Figure 17 illustrates the adopted fully connected architecture, which is composed of three hidden layers. Each hidden layer consists of Linear–ReLU–Batch Norm–Dropout layers, with 1000, 500 and 250 neurons, respectively. The hidden layer structure and the neuron size ratio are similar to the architecture used for training embeddings in e.g. Guo et al. (2016)^36,61,62^. The output layer is a linear layer with a sigmoid activation function.

For all RS2-InSAR datasets, all InSAR metadata are treated as categorical variables, except for one continuous variable: ”elapsed” (time). Therefore, embeddings will not be created for this continuous variable. The entities selected as categorical variables are listed in Tables 4 and 5 for RS2-InSAR descending and 2D decomposed motions, respectively. The entities for RS2-InSAR descending are the same as the ones created for the S1 descending data in Bayaraa et al. (2023)^36^.

The decomposed vertical and horizontal components of RS2-InSAR are combined into a unified dataset, rather than being handled separately by different models. This integration approach potentially offers the model additional hidden insights and relationships within the dataset that are not captured if the data are handled separately. An additional entity for the direction of the motion component, termed $motion\_comp$ is introduced within the RS2-InSAR vertical and horizontal stack to distinguish between them. It is treated as a categorical variable, as summarised in Table 5. The embedding size proposed in FastAI^62^ is adopted, where the embedding size equals $1.6 \gamma ^{0.52}$.

The EE-DL workflow described in Bayaraa et al. (2023)^36^ cannot be directly applied on RS2-InSAR. While the embedding size rule from Howard et al. (2020)^63^ works well for S1 InSAR, it requires some adjustment for RS2-InSAR. The embedding size rule in Howard et al. (2020)^63^ assigns a value of 600 to very high cardinality variables, which is potentially too large. Therefore, the embeddings for longitude and latitude are capped at 200. Other key variables that require adjustment include, an increase in the batch size from 1000 to 20,000 for ascending/descending, and 50,000 for the decomposed data. Moreover, the learning rate is adapted cyclically following Smith et al. (2017)^64^.

**Table 4:** Embeddings for RS2-InSAR descending. The embedding size is calculated based on the size of the cardinality of the entities. The cardinality is the number of unique categories representing each entity.

Entity	Cardinality	Embedding size
Longitude	224161	200
Latitude	224161	200
Day	12	6
Month	10	6
Year	3	3

**Table 5:** Embeddings for RS2-InSAR vertical and horizontal stack. The embedding size is calculated based on the size of the cardinality of the entities. The cardinality is the number of unique categories representing each entity.

Entity	Cardinality	Embedding size
Longitude	101813	200
Latitude	101813	200
Day	18	8
Month	9	5
Year	3	3
motion_comp	3	3

Due to the differing temporal resolutions of RS2 and S1 acquisitions, RS2-descending experiments within the EE-DL workflow generate predictions for dates ranging from 2018-01-10 to 2018-02-27 (’YYYY-MM-DD’). These dates are the closest matches to the EE-DL S1 prediction dates in Bayaraa et al. (2023)^36^. Given the less frequent acquisitions of RS2-InSAR, predictions are made for three steps instead of four steps (i.e. dates before failure). In contrast, the decomposed RS2-InSAR data, derived from the combination of ascending and descending acquisitions, offer a temporal resolution comparable to that of S1-InSAR. As a result, the last four dates closest to those in Bayaraa et al. (2023)^36^ are selected for prediction in these data sets.

Finally, the EE-DL framework employed in this study is inherently data-driven and does not explicitly encode physical laws or geotechnical constraints. Consequently, while the model is effective at learning spatio-temporal deformation patterns and identifying anomalous behaviour, its predictions should not be interpreted as deterministic forecasts of failure. Model performance is conditioned on the deformation regimes represented in the training data, therefore, previously unobserved mechanisms or abrupt changes in system behaviour may therefore be under-represented. In addition, the framework is sensitive to data availability and acquisition characteristics, including temporal sampling, orbit geometry, and data gaps, which can influence anomaly detectability. These limitations motivate the integration of the deep learning component with physics-based FE modelling and independent InSAR observations, which provide physical context and constraint for interpreting detected anomalies.

## Data Availability

The data that support the findings of this study are available from MDA Space, Terramotion Ltd. and Planet Labs but restrictions apply to the availability of these data, which were used under license for the current study, and so are not publicly available. Data are however available from the authors upon reasonable request and with permission of MDA Space, Terramotion Ltd. and Planet Labs.
